# The Shaftesbury Memorial

**Published:** 1888-10-13

**Authors:** 


### THE SHAFTESBURY MEMORIAL.

It is difficult to proclaim too often the message of a noble life. It is not difficult in this country to find such messages for proclamation. Our countrymen were reminded once more last week that a once-familiar voice and figure had ceased to take any share in the joys and sorrows of his brother men. The unveiling of the Shaftesbury Memorial in Westminster Abbey again pointed the story of an irreparable loss. Three years have elapsed since the great philanthropist, whose virtues gave lustre to an honoured name, passed beyond the ken of all who knew and trusted and loved him so well. No memorial was needed to preserve the memory of one whose whole life was made up of deeds which grave themselves on individual lives and on a country's history. No memorial can add to the worth of a character which was of the finest gold unmixed with baser clay. Those who raise memorials to such men as Lord Shaftesbury can confer no honour upon the dead; they can only satisfy an instinct and a feeling which not to satisfy would be to confess the feebleness of their own human virtues. But though no memorial at Westminster can add to the noble reputation of so noble a man, it can and will be felt by all his fellow-countrymen to be a fitting tribute, on their part, of honour and of an undiminished sense of loss.

